# Global human security in the post–COVID-19 era: The rising role of East Asia

**DOI:** 10.1371/journal.pmed.1003939

**Published:** 2022-07-14

**Authors:** Kenji Shibuya, Chorh Chuan Tan, Asaph Young Chun, Gabriel M. Leung

**Affiliations:** 1 Soma COVID Vaccination Medical Center, Soma City, Fukushima, Japan; 2 Yong Loo Lin School of Medicine, National University of Singapore, Singapore; 3 Statistics Research Institute | Statistics Korea, Daejeon, The Republic of Korea; 4 School of Public Health, LKS Faculty of Medicine, The University of Hong Kong, Hong Kong Special Administrative Region, People’s Republic of China; 5 WHO Collaborating Centre for Infectious Disease Epidemiology and Control, School of Public Health, LKS Faculty of Medicine, The University of Hong Kong, Hong Kong Special Administrative Region, People’s Republic of China; 6 Laboratory of Data Discovery for Health (D^2^4H), Hong Kong Science Park, New Territories, Hong Kong Special Administrative Region, People’s Republic of China

## Abstract

Kenji Shibuya and coauthors discuss the potential contribution of East Asian countries to global health in the light of COVID-19.

**Summary points**

- East Asia, comprising the 10 ASEAN countries, China, Japan, and the countries of the Korean peninsula, has achieved comparatively good outcomes during the ongoing Coronavirus Disease 2019 (COVID-19) pandemic.
- This can be explained by sociological imprinting of and learning from past outbreaks, as well as competent governance.
- Concomitantly, East Asian nations have also been expanding capacity in global health development and diplomatic outreach, although there is as yet no coherent regional bloc vision, shared strategy, or a common set of operating principles, thus limiting synergistic impact.
- We believe that concrete next steps to bolster cooperation and extend influence could include the establishment of an East Asian Center for Disease Control, joint work in health and human security by the Asian Development Bank (ADB) and Asian Infrastructure and Investment Bank, and a region-wide research funding programme.
- Much, however, depends on evolving geopolitics writ large, notably the instability and reorientation of global alliances, which have the potential to adversely affect relations between neighbouring East Asian member states.
- Health diplomacy for global human security has the potential to become a stabilising influence and can be a topic around which all actors can more comfortably rally.

While the constituent countries of East Asia share common elements of history and culture, there is great diversity and rapid transition in social systems, economic development, demography, and epidemiological profiles (S1 Table). These factors fundamentally lead to the full range of major global health challenges, including those concerning epidemics and pandemics of novel and reemerging pathogens.

Here, in the light of Coronavirus Disease 2019 (COVID-19), we discuss East Asia’s experience in this and past major outbreaks, its capacity and willingness to share best practice and support global health development, and the regional bloc’s potential in reshaping the global architecture for human security.

Geographically and ethnoculturally, East Asia has conventionally referred to the region comprising China, Japan, Republic of Korea (South Korea), Democratic People’s Republic of Korea (North Korea), and Mongolia. More recently, the term has been broadened to encompass Southeast Asia (viz the 10 member states of ASEAN, the Association of South East Asian Nations), largely due to expanded regional economic cooperation and latterly for geopolitical reasons.

## Imprinting of past outbreaks

**Fig 1:**
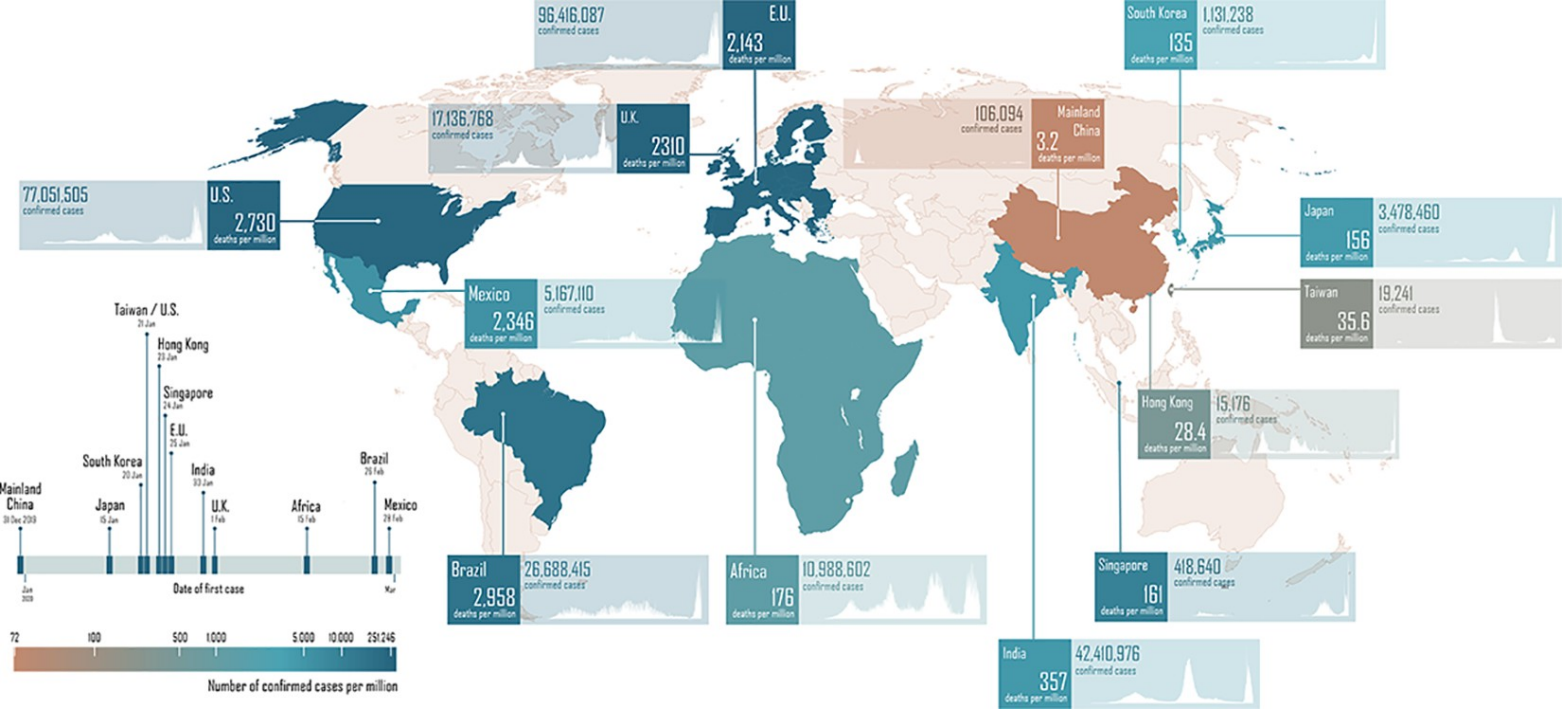
Comparative COVID-19 burden of disease as at January 11, 2022. (The map is in the public domain: https://commons.wikimedia.org/wiki/File:BlankMap-World.svg). COVID-19, Coronavirus Disease 2019.

COVID-19 has prompted reflections on how the world has come to the present state of the ongoing pandemic, drawing lessons from the past century of global outbreaks since the defining 1918 influenza pandemic [1]. Further, the heterogeneity of preparedness and response in different regions and countries during COVID-19 has motivated the search for explanations. For example, East Asia seems to have suffered a fraction of the global COVID-19 incidence and mortality burden so far, despite having hosted the original epicentre and first wave of cases (Fig 1).

S1 Fig shows a chronology of landmark epidemics and pandemics in the past 100 years. The 1957 Asian and 1968 Hong Kong influenza outbreaks eponymously locate the origin of the pandemics to East Asia. Severe Acute Respiratory Syndrome (SARS) and COVID-19 were first detected in the region, whereas South Korea experienced a large nosocomial outbreak of Middle East Respiratory Syndrome (MERS). Both avian–human outbreaks of H5N1 and H7N9 influenza A originated in China. Large outbreaks of hand, foot, and mouth disease occurred throughout East Asia, particularly since 2008. These directly transmissible respiratory pathogens of the past century have demonstrably and predominantly emerged from the region.

Therefore, East Asia may possess certain ecological and environmental characteristics that could have facilitated the emergence of novel respiratory viruses. The propensity for zoonotic spillovers is one major factor. For example, poultry supply chains in major producing nations, notably China and Vietnam in East Asia, as well as neighbouring Indonesia, still feature the long-entrenched live poultry market trade that remains responsible for half of all birds consumed in those countries. From farm production (including small, family-owned, “backyard” farms), to transport over long distances reaching wholesale markets, thence distribution to retail outlets, there are plenty of opportunities for virus amplification among the flocks and zoonotic events crossing the avian–human interface [2]. With respect to the latest outbreak of COVID-19, the first cluster was detected in a wholesale market selling seafood and many other items including wild game, although patient zero probably did not originate from that setting [3]. While the reservoir or source remains to be determined, the illicit pangolin trade has already been implicated as a possibility [4]. When human-to-human transmission becomes established, the possibility of spread has been raised by rapid urbanisation leading to large densely populated cities and the growth of transportation infrastructure allowing mass movement of people. Related to the better understanding of these fundamental driving forces of pathogen emergence is the issue of how East Asia and its governments should take greater responsibility for and enhance concerted efforts to prevent zoonoses and mitigate uncontrolled transmission in metropolitan settings through deliberate measures in urban planning.

In addition, the sociological imprints of multiple, repeated major emergence events might well have influenced population readiness and behaviour as well as governments’ predisposition to preparedness and response. In particular, SARS in 2002 to 2003 had left a deep impression on the collective psyche of East Asian countries that were affected: mainland China, Taiwan, Hong Kong Special Administrative Region, and Singapore. These and other East Asian governments revamped their surveillance and response infrastructure, built additional hospital isolation wards, and invested in workforce training, just as many European and American counterparts retrenched and pulled back funding support for these critical public health functions [5,6]. For example, China invested USD850 million after SARS to develop a new 3-tier system to prevent and control infectious diseases [7]. All the affected polities have significantly upgraded their health facilities, public health systems, and laboratory facilities for testing [8]. Importantly, large numbers of policy makers, health professionals, and frontline workers have learnt how to work together in outbreak management, within and between regional countries, and the majority of the public have first-hand experience of living through an epidemic.

At the individual level, personal hygiene practices and perception of what might be deemed appropriate and acceptable behaviour changed. Take mask wearing as an example: whether to don face masks or facial covering to prevent community spread has perhaps been one of the most argued and divisive issues in COVID-19, initially between East Asia and the West and later along partisan fissures within Western countries. Even the World Health Organization (WHO) had prevaricated on the issue during the initial months of the pandemic until it finally advised governments to “encourage the general public to wear masks in specific situations and settings as part of a comprehensive approach to suppress SARS-CoV-2 transmission” in its June 2020 guidance [9]. Another instance concerns sharing dishes at meals that is deeply embedded in East Asian food culture. The sustained and near universal adoption of serving utensils has been a relatively recent phenomenon since the time of SARS. Last, the tolerance, even willing acceptance of and thus adherence to, fairly draconian physical distancing and quarantine/isolation measures stand in stark contrast to an ever rising chorus of anti-maskers, anti-vaxxers, and anti-lockdown activists in many Western countries, particularly the United States [10,11].

## COVID-19 responses in East Asia

Comparing the COVID-19 responses in China, Hong Kong Special Administrative Region, Singapore, South Korea, and Taiwan, we identify 4 main East Asian characteristics that may be relevant to improving global preparedness and response to future outbreaks [8,12,13].

Chief among these is the critical importance of maintaining high levels of preparedness and the willingness and ability to respond swiftly and robustly to suppress an epidemic very early in its course. This has allowed for the policy goal of “near elimination” or “zero COVID” in all of these places. Crucial corequisites include sustained prior investment in strengthening public health infrastructure and capabilities; coordinated public health emergency management systems; clear structures to liaise across multiple agencies; a strong science base; effective testing, contact tracing, and isolation capabilities; stockpiling of personal protective equipment, medical supplies, and equipment; and good public communications built around comprehensive and transparently shared data. Countries that have not placed similar emphases, including those highly rated for epidemic preparedness before COVID-19 [14], have done less well in mitigating the worst effects of the current pandemic [15,16].

A second learning point from the East Asian COVID-19 response is the need to swiftly adapt strategies and innovate as more becomes known about the virus and how it spreads. For example, South Korea showed that very big outbreaks can be controlled or prevented through rapid high-volume testing of large numbers of potentially exposed individuals, obviating the need for a full national lockdown [17]. The initial motivation was to keep ahead of viral transmission. However, with the recognition that patients are most infectious at, and 2 to 3 days before, the onset of symptoms [18], this high-volume testing approach has become more salient as an important complement to contact tracing to prevent and reduce spread. In another example of a shift from the containment approach developed for SARS, Singapore rapidly deployed large-scale community facilities with telemonitoring capabilities to manage a big surge in mildly affected COVID-19 patients, thereby allowing hospitals to continue their regular operations.

Third, East Asia demonstrated how the extensive application of technology, data fusion, and analytics can substantially contribute to suppressing the spread of this highly infectious virus. For example, South Korea rapidly developed the 3-T scientific system of testing, tracing contacts, and treating in isolation, by leveraging “a walk-through testing system” that allows minimal contact with potentially infected people, tracing contacts based on artificial intelligence (AI)-based analysis of mobile big data and card transactions, and treating patients based on AI-driven analysis of clinical data [19]. Taiwan integrated its national health insurance database and immigration and customs database to enable big data analytics informing public health actions such as generating real-time alerts for clinicians and air travellers [20]. Hong Kong applied geofencing technology to enforce home quarantines through the use of a “StayHomeSafe” app and Bluetooth-paired wristband [21]. The combined use of 2 apps in Singapore, “TraceTogether” and “SafeEntry,” has halved the time taken to identify and quarantine close contacts from 4 days to less than 2 days [22].

**Fig 2:**
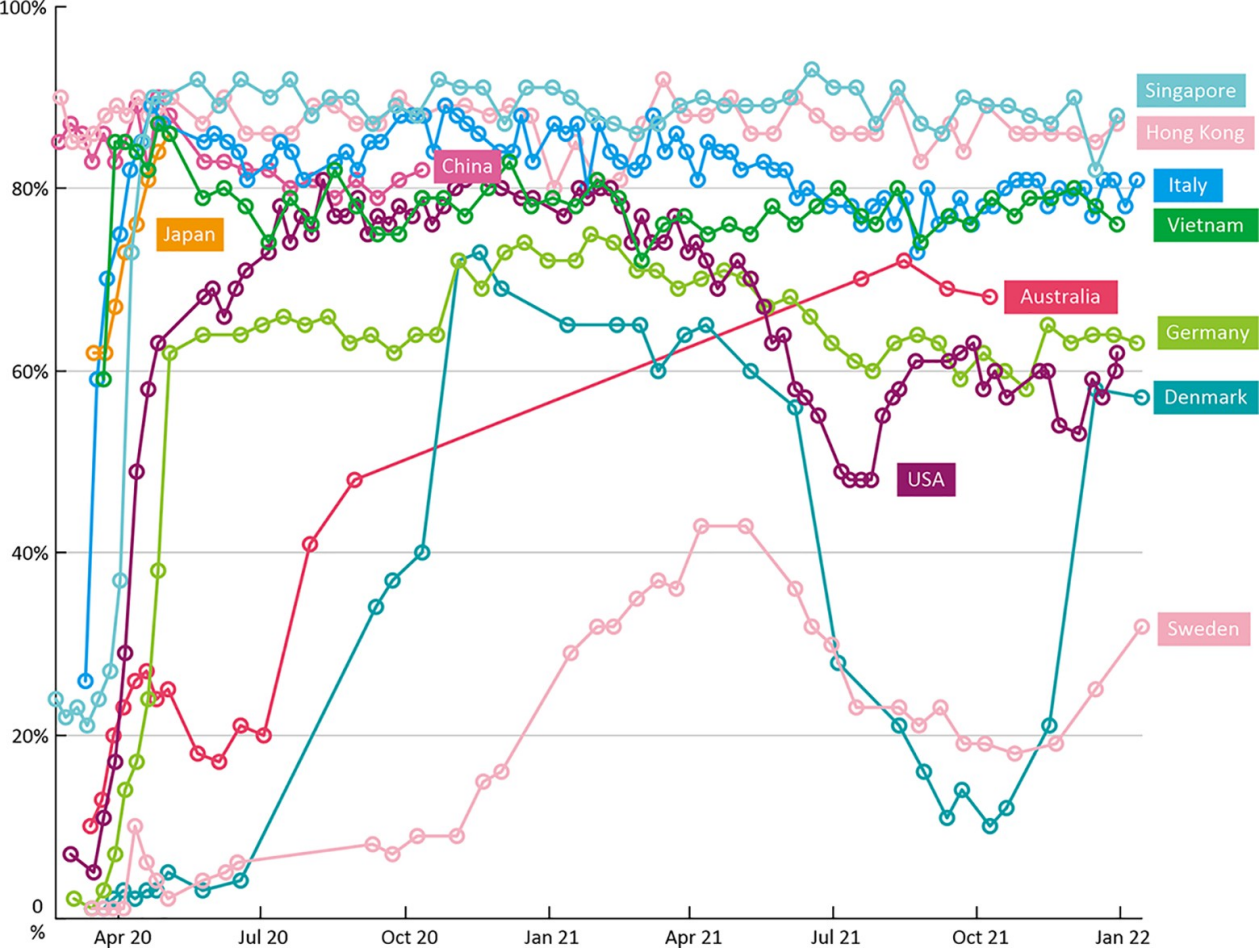
Face mask wearing in different populations. Source: https://yougov.co.uk/topics/international/articles-reports/2020/03/17/personal-measures-taken-avoid-covid-19.

Fourth, the COVID-19 pandemic has highlighted the importance of strong political leadership, coupled with a collective societal receptiveness to adhering to wide-ranging public health measures. The acceptance of universal masking in mainland China, Hong Kong, South Korea, and Japan exemplifies this, especially as this was widely practiced before the importance of masking as source control to reduce asymptomatic spread, was broadly accepted [23] (Fig 2). Recent research demonstrates high correlation between collectivism and mask usage [24]. Research following the 2003 SARS epidemic indicated that people understood and accepted the need for restrictive measures and were willing to sacrifice their right to freedom of movement [25], and this appears to have persisted. However, as the public health interventions increasingly use data and technologies to detect, contain, and prevent infection clusters, issues relating to data privacy, confidentiality, and security will come to the fore, and could, over time, erode the acceptability of these measures [26]. It is important, therefore, in the design and deployment of such technologies, that every care is taken to address and mitigate these increasingly popular concerns.

Notwithstanding these relative successes, lessons from East Asia should be understood in context. In addition to experiential learning through sociological imprinting by past outbreaks, East Asian societies tend to value collective well-being sometimes at the expense of individual liberties. Preexisting social structures to enforce mass lockdowns are difficult to replicate elsewhere. Taking the mainland Chinese example, containment had been greatly facilitated by neighbourhood-level organisations that monitored home quarantine and self-medical surveillance. It was further buttressed by the ubiquitous penetration of social and payment apps that had been quickly adapted for contact tracing purposes and as mobility passports. Finally, while a “zero COVID” approach might have saved tens or even hundreds of thousands of lives in 2020, this success may have paradoxically retarded vaccine acceptance despite adequate access and poses an ongoing conundrum of how best to reopen society.

## Health diplomacy and development assistance: The rapidly changing role of East Asia

Until the 1990s, Japan had been the only major donor in East Asia, and the majority of East Asian economies had been recipients of development assistance for health (DAH). This has changed substantially and rapidly since the mid-2000s, where China and South Korea have been increasing the size and scope of DAH (S2 Table). This trend is grounded in both an altruistic concern for the most vulnerable populations, and an understanding that in today’s interconnected, globalised world, a health crisis in any country can impact their own populations. Accordingly, national interests associated with global health have shifted from the development context to cover both economic and security perspectives [27]. Likewise, the scope of global health has broadened from delivery of services to include discovery and development of interventions [28].

Beyond aid, it would be important to consider the overall global health architecture, defined as “the relationship between the many different actors engaged in global health and the processes through which they work together” [29]. In recent years, debates about this have intensified, largely driven by the complex interactions between health transitions and global health priorities, as well as uncertainties in global governance and economic prospects [30]. These challenges are further confounded by new and emerging actors and stakeholders.

To date, DAH in East Asia has primarily been driven by government-controlled, Overseas Development Aid (ODA)-focused development agencies such as the Japan International Cooperation Agency (JICA), Korea International Cooperation Agency (KOICA), and latterly China International Development Cooperation Agency (CIDCA) since 2018. Regional multilaterals, primarily led by seconded officials from Japan and South Korea, such as WHO’s Western Pacific Regional Office and the Asian Development Bank (ADB) also play an important role (S2 Table). China launched the Asian Infrastructure Investment Bank (AIIB) in 2015 as a complement or alternative to ADB and even the World Bank. Japan, South Korea, and China also influenced global health policy by successfully nominating their nationals to become WHO Director-General during 1988 to 1998, 2003 to 2006, and 2006 to 2017, respectively. Although the number of nongovernmental organisations in global health has increased in the region in recent years, including the International Vaccine Institute in South Korea, the contributions from civil society and the nonpublic sector in East Asia to regional and global health remain generally limited and fragmented.

In terms of support, Japan’s ODA is guided by the view of assistance as part of a partnership with recipient countries. Japan has prioritised its health ODA in capacity building and sustainability, which is unique among major donors [31]. With few exceptions, Japan’s emphasis on self-reliance has also kept its government from providing general, unspecified budget support to the recipient country’s health sector—a practice that has become popular among European donors [32]. Japan also used G7/8 Summits to influence global health policy including establishing the Global Fund to Fight AIDS, Tuberculosis and Malaria (2000), strengthening health systems and universal health coverage (2008), and revamping health security systems (2016) [33]. Of pertinence to COVID-19, Japan, together with Norway, Germany, the Wellcome Trust, and Bill and Melinda Gates Foundation, was among the initial group of major donors to the Coalition for Epidemic Preparedness Innovation (CEPI) that is playing a critical role in developing effective vaccines [34]. That said, Western governments and philanthropies have remained the intellectual and financial pillars of the Coalition.

Global health has also been a priority issue in South Korea’s development policy since its inclusion in the second Strategic Plan for International Development Cooperation for 2016 to 2020. Its goal is to contribute to the achievement of universal health objectives by improving access to quality health and medical services and care for all [35]. South Korea’s DAH has increased substantially since 2000, and more investment in global health is expected as South Korea increases its ODA spending to meet the government’s 0.3% of gross national income (GNI) target [36].

In 2013, China launched the Belt and Road Initiative (BRI) to promote trade, infrastructure, and commercial relations with 65 countries in Asia, Africa, and Europe. This initiative contains important health components. China’s DAH has so far been primarily deployed to develop infrastructure, dispatch medical personal, and provide medical supplies to Africa and Asia [37]. In recent years, however, China has become an emerging donor country for global health in East Asia (S2 Table) and supplying its vaccines to low- and middle-income countries directly and through the existing global architecture including the COVAX facility. However, it is not a financial contributor to COVAX. COVAX is a multilateral platform to which more than 180 countries have subscribed, although it has not entirely lived up to its aspired goal of ensuring equitable vaccine access to date.

As a group, however, thus far there has been little regional coherence, or tendency to foster such, among the bigger players in North East Asia (China, Japan, and South Korea) nor within ASEAN, let alone region wide. Where bilateral actions currently predominate, there remains much room to bring synergistic impact through multilateralism, perhaps in a different way than the post-WWII institutions currently operating. However, a shared vision that can be operationalised through a set of common principles and goals would be prerequisites.

Of course, it would be naive to understand DAH in isolation from any country’s foreign policy, projection of its soft power, and geopolitics writ large. The increasingly patent bipolar reorientation of global alliances around China (BRI, ASEAN-initiated Regional Comprehensive Economic Partnership, and Shanghai Cooperation Organisation) and the US (“The Quad” and “Five Eyes [38]”, D10 [39], NATO, etc.), with Europe and the United Kingdom playing a pivotal role in the balance of power, particularly at the time of COVID-19 pandemic, will directly as well as insidiously sway and frame the dynamic nature of health diplomacy and development aid policies.

## Opportunities and responsibilities for the future

At the risk of over-generalising internally heterogeneous regions, the clear disparities in COVID-19 outcomes between East Asia and much of the West have prompted some to invoke regional cultural exceptionalism as an explanation. Rather than this, the more salient factor is one of good epidemic governance—defined by the Independent Panel for Pandemic Preparedness and Response as “instituting whole-of-government approaches with clearly defined, tiered command structures to prepare for and respond to future outbreaks, with clear involvement of communities and transmission of information. Health protection functions were consolidated under new centralised agencies [40].” Of note, the performance of Australasia to date, through its “zero COVID” approach, generally considered to be part of the West but having achieved COVID outcomes similar to or better than East Asian nations, further undermines the simplistic cultural determinants explanation.

That said, with East Asian governments having demonstrated sustained commitment to, and competence in, pandemic control, Kishore Mahbubani was unequivocal in asserting that “deference to Western societies, which was the norm in the 19th and 20th centuries, will be replaced by a growing respect and admiration for East Asian ones. The pandemic could thus mark the start of the Asian century [41].” One could therefore imagine that East Asia should progressively bear greater responsibility in sharing and spreading best practices towards securing a common healthy future with and for the world. Indeed the process has already begun before COVID-19 and is built on a burgeoning track record of global health engagement.

Japan’s long-standing emphasis on human security as a guiding principle for global health, coupled with its strong leadership of espousing universal health coverage as a bedrock principle for advancing sustainable development, has long been an important engine for the world [42]. More recently, China’s health diplomacy forays, such as bilateral assistance in the building of laboratories and treatment facilities during the 2013 to 2016 West Africa Ebola outbreak [43] or pledging to deliver at least 2 billion doses of Chinese COVID-19 vaccines to COVAX, form a solid lattice on which to build future efforts. On the conference circuit of global thought leadership, we have been witnessing an accelerating trajectory among East Asian hosts. A case in point is the China-led Boao Forum for Asia, which started in 2001 and established a standalone global health forum since 2019. Singapore’s annual Raffles Dialogues on Human Well-Being and Security is another. Thailand’s Prince Mahidol Award Conference has long been a global health fixture. These have provided different and complementary perspectives to those emanating from the Western hemisphere, including the World Economic Forum (Davos, Switzerland) and the World Health Summit (Berlin, Germany) [44]. Regional joint learning hubs like the Thai International Health Policy Program, the global health programmes and schools of public health at the Universities of Tokyo and Hong Kong and the National University of Singapore, regional initiatives such as the Asia Pacific Observatory on Health Systems and Policies, and East Asian–based philanthropies like the China Medical Board provide intellectual underpinnings to such thought leadership.

East Asian countries have therefore been ramping up diplomatic efforts around the health axis. With quickening shifts in the geopolitical tectonic plates, notably Sino-American tensions and the consequential ripple effects on allies and developmental aid recipients, health diplomacy for global human security has the potential to become a stabilising influence and can be a topic around which all global actors can more comfortably rally. Strategies that should be considered are listed in Box 1, many of which have been reprised by the Independent Panel [40].

**Box 1. Six key actions towards securing a common healthy future**

1. Reforming WHO, including adequate and sustainable financing of core operations
2. Reimagining the International Health Regulations and their adherence, including enforcement mechanisms
3. Realigning the roles and responsibilities of other multilateral players, including the development banks and philanthropies
4. Redressing disparities in disease burden and means to mitigate outbreaks between and within countries/regions
5. Reinforcing national health system preparedness for epidemics and holding governments accountable
6. Reinvigorating discovery research and reconnecting research with implementation

While the WHO Western Pacific Regional Office is its most heterogeneous in terms of development needs and size of member states, the constituent East Asian anchors have provided steady and competent leadership support to steer the agency in fulfilling its mandate with good success over decades. This experience and role should be extended to the global context as WHO finds a new and sustainable role in the post–COVID-19 world. CEPI and to a lesser extent COVAX have emerged as useful collaboratives for global public good and in the ongoing pandemic. They could be templates to consider how multilateral financing mechanisms should be made to really work for outbreak preparedness and response in future. Similarly, the philanthropic models of the West, like the Wellcome Trust and the Gates Foundation, should be joined by rising wealth in East Asia in synergy. Generally, if German science gave the world its first fruits of life science discoveries, and Anglo-American research our present prowess, surely our ability to address the most pressing global health issues will be greatly increased by the leadership and contributions of Chinese, Japanese, Korean, and other East Asian scientists whose accelerated productivity in terms of scientific papers and intellectual property has been impressive [45].

More concretely, next steps could include the establishment of a Center for Disease Control for the region, after the European and African models. ADB’s recent reentry into and AIIB’s exploration of the health space could be useful preludes to a joint effort. A region-wide research funding programme is another possibility to bolster cooperation and collaboration. These would all create opportunities to engage with other regional counterparts and thus spread influence.

In conclusion, East Asian nations bear an added set of responsibilities towards global human security in the post–COVID-19 era. The pivoting of such a leadership opportunity towards the East preceded the current pandemic but has been hastened by it. The commendable performance, so far, of the region in mitigating the worst impact of COVID-19 can be explained by its developmental trajectory, imprinting by and learning from past outbreaks, without needing to resort to “Asian values” or cultural separatism. In *Development as Freedom*, Amartya Sen disputed the idea that Asian values are quintessentially collectivist and authoritarian, or for that matter, Western values have always attached great value to individual freedom [46]. Therefore, rather than accentuating the contextual differences between East Asia and the rest of the world, in attempting to explain and replicate the former’s recent successes in human security, we should focus on embracing diversity in circumstances and histories by presuming commonalities. This way we could better harness the burgeoning health diplomatic outreach of East Asia amid shifting geopolitics and chart a new course towards a common, secure, and healthy future of globalism 2.0.

## Supporting information

S1 Fig Chronology of major pandemics and epidemics in the past century. (TIF)

S1 Table Basic demographic, economic, and epidemiological indicators in East Asia. (DOCX)

S2 Table DAH by source of funding among East Asian donors, 1990 to 2019 (USD in millions). DAH, development assistance for health.
(DOCX)

## Abbreviations

ADB: Asian Development Bank
AI: artificial intelligence
AIIB: Asian Infrastructure Investment Bank
BRI: Belt and Road Initiative
CEPI: Coalition for Epidemic Preparedness Innovation
CIDCA: China International Development Cooperation Agency
COVID-19: Coronavirus Disease 2019
DAH: development assistance for health
GNI: gross national income
JICA: Japan International Cooperation Agency
KOICA: Korea International Cooperation Agency
MERS: Middle East Respiratory Syndrome
ODA: Overseas Development Aid
SARS: Severe Acute Respiratory Syndrome
WHO: World Health Organization

## References

[1] Morens DM, Fauci AS. Emerging Pandemic Diseases: How We Got to COVID-19. Cell. 2020;182(5):1077–92. doi: 10.1016/j.cell.2020.08.021 32846157 PMC7428724

[2] Peiris JS, Cowling BJ, Wu JT, Feng L, Guan Y, Yu H, et al. Interventions to reduce zoonotic and pandemic risks from avian influenza in Asia. Lancet Infect Dis. 2016;16(2):252–8. doi: 10.1016/S1473-3099(15)00502-2 26654122 PMC5479702

[3] Li Q, Guan X, Wu P, Wang X, Zhou L, Tong Y, et al. Early Transmission Dynamics in Wuhan, China, of Novel Coronavirus-Infected Pneumonia. N Engl J Med. 2020;382(13):1199–207. doi: 10.1056/NEJMoa2001316 31995857 PMC7121484

[4] Lam TT, Jia N, Zhang YW, Shum MH, Jiang JF, Zhu HC, et al. Identifying SARS-CoV-2-related coronaviruses in Malayan pangolins. Nature. 2020;583(7815):282–5. doi: 10.1038/s41586-020-2169-0 32218527

[5] McKee M, Gill M, Wollaston S. Public inquiry into UK’s response to covid-19. BMJ. 2020;369:m2052. doi: 10.1136/bmj.m2052 32444349

[6] Maani N, Galea S. COVID-19 and Underinvestment in the Public Health Infrastructure of the United States. Milbank Q. 2020;98(2):250–9. doi: 10.1111/1468-0009.12463 32333418 PMC7296430

[7] Bouey J. Strengthening China’s Public Health Response System: From SARS to COVID-19. 2020;110(7):939–40. 10.2105/AJPH.2020.305654 PMC7287547 32213081

[8] An BY, Tang SY. Lessons From COVID-19 Responses in East Asia: Institutional Infrastructure and Enduring Policy Instruments. Am Rev Public Adm. 2020;50(6–7):790–800.

[9] World Health Organization. Advice on the use of masks in the context of COVID-19: interim guidance, 5 June 2020. World Health Organization; 2020.

[10] Yong E. How the pandemic defeated America. A virus has bought the world’s most powerful country to its knees. [cited 2020 Oct 18]. Available from: https://www.theatlantic.com/magazine/archive/2020/09/coronavirus-american-failure/614191/?utm_term=2020-08-03T22%3A51%3A34&utm_content=edit-promo&utm_medium=social&utm_source=twitter&utm_campaign=the-atlantic.

[11] Hsiang S, Allen D, Annan-Phan S, Bell K, Bolliger I, Chong T, et al. The effect of large-scale anti-contagion policies on the COVID-19 pandemic. Nature. 2020;584(7820):262–7. doi: 10.1038/s41586-020-2404-8 32512578

[12] Lu N, Cheng KW, Qamar N, Huang KC, Johnson JA. Weathering COVID-19 storm: Successful control measures of five Asian countries. Am J Infect Control. 2020;48(7):851–2. doi: 10.1016/j.ajic.2020.04.021 32360746 PMC7189844

[13] Shaw R, Kim YK, Hua J. Governance, technology and citizen behavior in pandemic: Lessons from COVID-19 in East Asia. Prog Disaster Sci. 2020;100090. doi: 10.1016/j.pdisas.2020.100090 34171010 PMC7194878

[14] Nuclear Threat Initiative (NTI), Johns Hopkins Center for Health Security (JHU), Economist Intelligence Unit (EIU). Global Health Security Index. [cited 2020 Oct 17]. Available from: https://www.ghsindex.org/about.

[15] Scally G, Jacobson B, Abbasi K. The UK’s public health response to covid-19. BMJ. 2020;369:m1932. doi: 10.1136/bmj.m1932 32414712

[16] Xu HD, Basu R. How the United States flunked the COVID-19 test: some observations and several lessons. Am Rev Public Adm. 2020;50(6–7):568–76.

[17] Dighe A, Cattarino L, Cuomo-Dannenburg G, Skarp J, Imai N, Bhatia S, et al. Response to COVID-19 in South Korea and implications for lifting stringent interventions. BMC Med. 2020;18(1):321. doi: 10.1186/s12916-020-01791-8 33032601 PMC7544529

[18] He X, Lau EH, Wu P, Deng X, Wang J, Hao X, et al. Temporal dynamics in viral shedding and transmissibility of COVID-19. Nat Med. 2020;26(5):672–5. doi: 10.1038/s41591-020-0869-5 32296168

[19] The Government of the Republic of Korea. Flattening the curve on COVID-19: How Korea responded to a pandemic using ICT. 2020 [cited 2022 Jan 11]. https://overseas.mofa.go.kr/gr-en/brd/m_6940/view.do?seq=761548&srchFr=&amp%253BsrchTo=&amp%253BsrchWord=&amp%253BsrchTp=&amp%253Bmulti_itm_seq=0&amp%253Bitm_seq_1=0&amp%253Bitm_seq_2=0&amp%253Bcompany_cd=&a

[20] Wang CJ, Ng CY, Brook RH. Response to COVID-19 in Taiwan: Big Data Analytics, New Technology, and Proactive Testing. JAMA. 2020;323(14):1341–2. doi: 10.1001/jama.2020.3151 32125371

[21] Huang Y, Sun M, Sui Y. How digital contact tracing slowed COVID-19 in East Asia. Harv Bus Rev. 15 April, 2020.

[22] Ministry of Health, Singapore. TraceTogether and SafeEntry to be enhanced in preparation for further opening of the economy. [cited 2020 Oct 17]. Available from: https://www.moh.gov.sg/news-highlights/details/tracetogether-and-safeentry-to-be-enhanced-in-preparation-for-further-opening-of-the-economy.

[23] YouGov. International COVID-19 tracker update: 8 June. Latest round-up of YouGov’s coronavirus survey results. [cited 2020 Oct 27]. Available from: https://yougov.co.uk/topics/international/articles-reports/2020/06/08/international-covid-19-tracker-update-8-june?fbclid=IwAR0ZMQVDthkM-eDDdpqrLkQMM6unpg5SN5O80_xh7yIDnqdGtyM13LqQY6M.

[24] Lu JG, Jin P, English AS. Collectivism predicts mask use during COVID-19. Proc Natl Acad Sci U S A. 2021;118(23):e2021793118. doi: 10.1073/pnas.2021793118 34016707 PMC8201834

[25] Blendon RJ, DesRoches CM, Cetron MS, Benson JM, Meinhardt T, Pollard W. Attitudes toward the use of quarantine in a public health emergency in four countries. Health Aff (Millwood). 2006;25(2):w15–25. 16434437 10.1377/hlthaff.25.w15

[26] Cha S, Smith J. South Korea to boost coronavirus tracing privacy amid fears of backlash: Reuters. [cited 2020 May 14]. Available from: https://www.reuters.com/article/us-health-coronavirus-southkorea-idUSKBN22Q0GD.

[27] Institute of Medicine Committee on the US Commitment to Global Health. The US commitment to global health: recommendations for the public and private sectors: National Academies Press (US); 2009. 20662131

[28] Kickbusch I, Silberschmidt G, Buss P. Global health diplomacy: the need for new perspectives, strategic approaches and skills in global health. Bull World Health Organ. 2007;85(3):230–2. doi: 10.2471/blt.06.039222 17486216 PMC2636243

[29] Kickbusch I, Lister G, Told M, Drager N. Global health diplomacy: Concepts, issues, actors, instruments, fora and cases. New York: Springer; 2012.

[30] Sakamoto H, Ezoe S, Hara K, Sekitani Y, Abe K, Inada H, et al. Japan’s contribution to making global health architecture a top political agenda by leveraging the G7 presidency. J Glob Health. 2018;8(2):020313. doi: 10.7189/jogh.08.020313 30546866 PMC6269922

[31] Ministry of Foreign Affairs of Japan. Japan’s official development assistance charter: revision of Japan’s Official Development Assistance Charter (August 2003). [cited 2020 Nov 1]. Available from: http://www.mofa.go.jp/policy/oda/reform/charter.html.

[32] Lancet T. International Health Partnership: a welcome initiative. Lancet. 2007;370(9590):801. doi: 10.1016/S0140-6736(07)61387-7 17826149

[33] Japan Global Health Working Group. Protecting human security: proposals for the G7 Ise-Shima summit in Japan. Lancet. 2016;387(10033):2155–62. doi: 10.1016/S0140-6736(16)30177-5 27301827

[34] Roberts RB. CEPI—A global partnership. Germs. 2017;7(1):8–9. doi: 10.18683/germs.2017.1102 28331836 PMC5348218

[35] KOICA. Mid-term sectoral strategy 2016–2020. [cited 2020 Oct 24]. Available from: http://67.199.83.28/doc/10.pdf.

[36] Committee for International Development Cooperation (CIDC). Second Mid-term Strategy for International Development Co-operation (2016–2020), November 10, 2015.

[37] Tang K, Li Z, Li W, Chen L. China’s Silk Road and global health. Lancet. 2017;390(10112):2595–601. doi: 10.1016/S0140-6736(17)32898-2 29231838 PMC7159269

[38] Mahbubani K. Why Attempts to Build a New Anti-China Alliance Will Fail—The big strategic game in Asia isn’t military but economic. 2021 [cited 2021 May 21]. Available from: https://foreignpolicy.com/2021/01/27/anti-china-alliance-quad-australia-india-japan-u-s/.

[39] Brattberg E, Judah B. Forget the G-7, Build the D-10—The moment is right for a summit of democracies. 2020 [cited 2021 May 21]. Available from: https://foreignpolicy.com/2020/06/10/g7-d10-democracy-trump-europe/.

[40] The Independent Panel for Pandemic Preparedness and Response. COVID-19: Make it the Last Pandemic. [cited 2021 May 21]. Available from: https://theindependentpanel.org/wp-content/uploads/2021/05/COVID-19-Make-it-the-Last-Pandemic_final.pdf. 10.1016/S0140-6736(21)01095-3 PMC9751704 33991477

[41] The Economist. Kishore Mahbubani on the dawn of the Asian century. 20 April, 2020.

[42] Abe S. Japan’s vision for a peaceful and healthier world. Lancet. 2015;386(10011):2367–9. doi: 10.1016/S0140-6736(15)01172-1 26700515 PMC7138109

[43] Huang Y. China’s Response to the 2014 Ebola Outbreak in West Africa. Global Chall. 2017;1(2):1600001. 10.1002/gch2.201600001 PMC6607196 31565261

[44] Pang T, Chong YS, Fong H, Harris E, Horton R, Lee K, et al. Yes we can! The Raffles Dialogue on Human Wellbeing and Security. Lancet Glob Health. 2015;3(8):E496–500. doi: 10.1016/S2214-109X(15)00102-3 26187492

[45] Nature Index 2020 Annual Tables 2020. [cited 2020 Oct 21]. Available from: https://www.nature.com/collections/chdeajdica.

[46] Sen A. Development as freedom. 2000; New York: Anchor Books. p. 230–48.

