# Supplementary figures and images for: Global human security in the post–COVID-19 era: The rising role of East Asia
Source: PLoS Med. 2022 Jul 14;19(7):e1003939. doi: 10.1371/journal.pmed.1003939 (PMC9282514; doi:10.1371/journal.pmed.1003939)

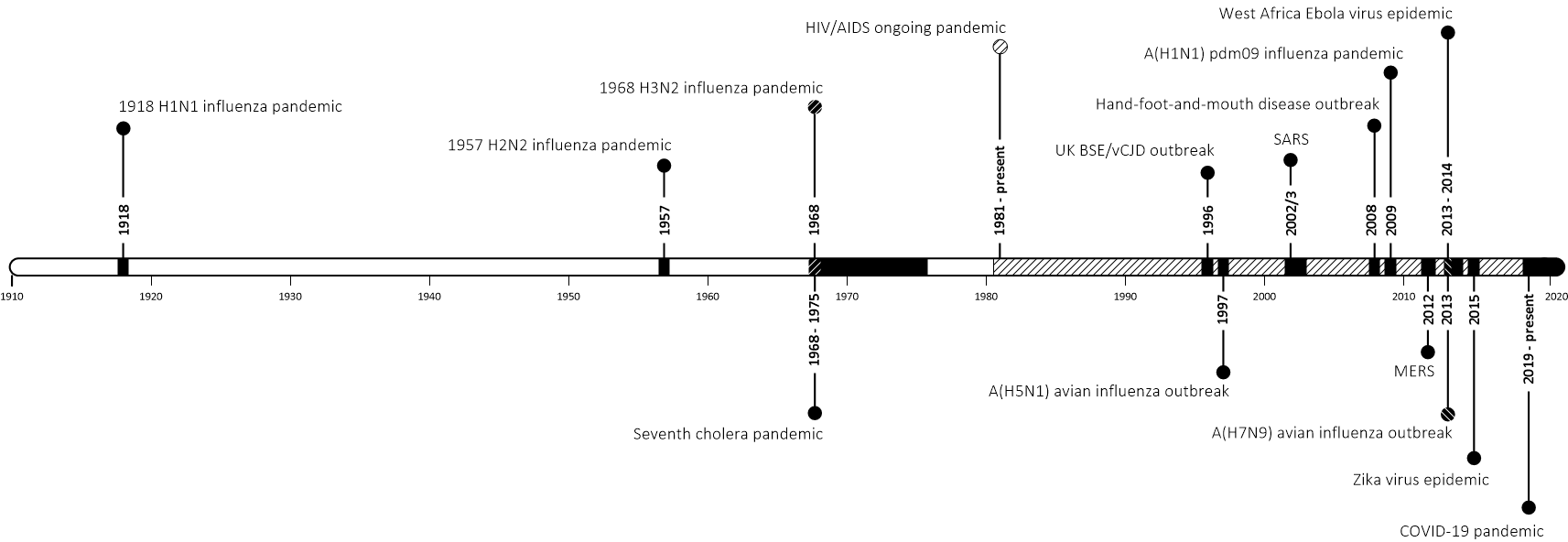

Supplement: S1 Fig — (TIF) [file pmed.1003939.s001.tif]
