# Supplementary material for: Global human security in the post–COVID-19 era: The rising role of East Asia
Source: PLoS Med. 2022 Jul 14;19(7):e1003939. doi: 10.1371/journal.pmed.1003939 (PMC9282514; doi:10.1371/journal.pmed.1003939)
Supplement: S1 Table — (DOCX) [file pmed.1003939.s002.docx]

**S1 Table. Basic demographic, economic and epidemiological indicators in East Asia**

| Country | Population (millions) | Population aged 65 years or over (%) | Life expectancy (years) | | Economy | | Development assistance for health (USD) | COVID-19 burden (as at January 11, 2022) | |
| --- | --- | --- | --- | --- | --- | --- | --- | --- | --- |
|  |  |  | Male | Female | Gross domestic product per capita (USD) | Health expenditure per capita (USD) |  | Reported cases | Reported deaths |
| Brunei Darussalam | 0.4 | 3.9 | 73.3 | 77.5 | 28,572 | 690 |  | 15,634 | 59 |
| Cambodia | 16.1 | 5.1 | 66.8 | 72.8 | 1,384 | 83 |  | 120,621 | 3,015 |
| China | 1,410.8 | 11.4 | 79.9 | 74.5 | 8,612 | 455 | 734.7 | 134,152 | 5,699 |
| Hong Kong SAR | 7.4 | 16.5 | 81.9 | 87.6 | 46,733 | 3,053 |  | 12,809 | 213 |
| Taiwan | 23.6 | 13.9 | 77.3 | 83.7 | 24,390 | 1,477 | 0.2 | 17,463 | 850 |
| Democratic People's Republic of Korea (North Korea) | 26.1 | 9.8 | 68.5 | 74.8 | 683 | 77 |  | 0 | 0 |
| Indonesia | 256.0 | 5.6 | 69.1 | 73.9 | 3,837 | 120 |  | 4,266,649 | 144,136 |
| Japan | 128.6 | 27.7 | 80.9 | 87.0 | 38,214 | 4,290 | 1172.4 | 1,765,604 | 18,403 |
| Lao People's Democratic Republic | 7.0 | 4.1 | 65.0 | 70.3 | 2,424 | 58 |  | 118,880 | 437 |
| Malaysia | 30.6 | 6.4 | 72.5 | 77.2 | 10,118 | 409 |  | 2,786,219 | 31,678 |
| Mongolia | 3.3 | 3.9 | 64.7 | 73.7 | 3,672 | 162 |  | 395,147 | 2,001 |
| Myanmar | 53.8 | 6.1 | 65.0 | 72.2 | 1,256 | 52 |  | 532,062 | 19,291 |
| Philippines | 108.6 | 4.8 | 66.6 | 73.2 | 2,982 | 133 |  | 2,965,447 | 52,150 |
| Republic of Korea (South Korea) | 52.7 | 13.6 | 79.5 | 85.5 | 29,958 | 2,118 | 348.8 | 667,390 | 6,071 |
| Singapore | 5.6 | 10.6 | 81.2 | 87.4 | 56,746 | 2,739 | 1.7 | 285,647 | 838 |
| Thailand | 69.8 | 11.4 | 73.9 | 81.3 | 6,579 | 271 |  | 2,277,476 | 21,838 |
| Viet Nam | 95.1 | 6.5 | 70.0 | 79.2 | 2,366 | 135 |  | 1,899,575 | 34,319 |
| Sources: GBD 2019 Demographics Collaborators (2020); Global Burden of Disease Collaborative Network (2020); WHO COVID-19 Dashboard; Centre for Health Protection, Department of Health, The Government of the Hong Kong SAR; Taiwan Centers for Disease Control | | | | | | | | | |
| * Census and Statistics Department, Hong Kong SAR. Hong Kong Population Projections 2017-2066; Hong Kong Domestic Health Accounts 2018/9 | | | | | | | | | |
