# Supplementary material for: Global human security in the post–COVID-19 era: The rising role of East Asia
Source: PLoS Med. 2022 Jul 14;19(7):e1003939. doi: 10.1371/journal.pmed.1003939 (PMC9282514; doi:10.1371/journal.pmed.1003939)
Supplement: S2 Table — DAH, development assistance for health. (DOCX) [file pmed.1003939.s003.docx]

**S2 Table. Development assistance for health by source of funding amongst East Asian donors, 1990-2019 (USD in millions)**

| **Donor** | **1990** | **1995** | **2000** | **2005** | **2010** | **2015** | **2019** |
| --- | --- | --- | --- | --- | --- | --- | --- |
| China | 82.8 | 98.8 | 142.1 | 217.5 | 443.2 | 585.9 | 734.7 |
| Taiwan | - | 0.2 | 0.1 | 0.4 | 1.1 | 0.5 | 0.3 |
| Japan | 617.6 | 964.9 | 933.0 | 856.3 | 1094.3 | 1031.0 | 1172.4 |
| Republic of Korea (South Korea) | 1.3 | 15.7 | 96.9 | 142.8 | 210.6 | 278.3 | 348.8 |
| Singapore | 0.6 | 0.9 | 1.0 | 2.3 | 2.3 | 2.6 | 0.1 |
|  |  |  |  |  |  |  |  |
| Asian Development Bank (ADB) | 1.3 | 98.7 | 56.1 | 192.9 | 334.4 | 115.2 | 320.7 |
| Note: All figures are in millions of of 2019 US dollars. | | | | | | | |
| Source: Global Burden of Disease Collaborative Network. Global Health Spending 2018-2050. Seattle: Institute for Health Metrics and Evaluation (IHME), 2020. | | | | | | | |
